# CYP4F22-Related Autosomal Recessive Congenital Ichthyosis: Clinical Presentation

**DOI:** 10.7759/cureus.22272

**Published:** 2022-02-16

**Authors:** Andrea Dumenigo, Amanda Rusk, Kalyani Marathe

**Affiliations:** 1 Internal Medicine, Philadelphia College of Osteopathic Medicine, Philadelphia, USA; 2 Pediatric Dermatology, Cincinnati Children's Hospital Medical Center, Cincinnati, USA

**Keywords:** cyp4f22- related congenital ichthyosis, collodion membrane, abnormal keratinization, cyp4f22, congenital ichthyosis

## Abstract

Autosomal recessive congenital ichthyosis (ARCI) is a group of hereditary, nonsyndromic disorders of keratinization. ARCI encompasses several different clinical presentations and is caused by various genetic mutations. Commonly, ARCI presents with a taut, thick, shiny stratum corneum called a collodion membrane, but patients with mutations in CYP4F22 frequently present only with erythroderma. We report the case of a patient who was heterozygous for a pathogenic variant and a variant of uncertain significance in the CYP4F22 gene and presented with a collodion membrane and developed a mild ichthyosis phenotype.

## Introduction

The term autosomal recessive congenital ichthyosis (ARCI) refers to a group of rare, nonsyndromic disorders of keratinization [1]. In the US, the prevalence of ARCI has been reported to be 1:200,000-300,000 [2]. ARCI is associated with mutations in various genes, including ABCA12, ALOX12B, ALOXE3, CYP4F22, NIPAL4, TGM1, CERS3, PNPLA1, CASP14, SDR9C7, AND SULT2B1 [3]. Most of these identified genes participate in the synthesis of enzymes and transporters involved in the construction, transport, and/or assembly of components of the stratum corneum [4]. A TGM1 gene mutation is the most common cause of ARCI, and it is strongly associated with a collodion membrane at birth [1]. Only 8% of ARCI cases are linked to a CYP4F22 mutation, making it extremely rare [1]. ARCI may present at birth with a shiny, taut membrane formed by a thickened stratum corneum, known as a collodion membrane [5]. Though this is a common clinical manifestation of ARCI, patients with a CYP4F22 mutation typically present with erythroderma, instead of a collodion membrane [5]. If present, the collodion membrane will dry up, crack, and peel off over several weeks, revealing the underlying ARCI phenotype. The common phenotypes include lamellar ichthyosis (LI) and congenital ichthyosiform erythroderma (CIE), but self-improving collodion ichthyosis (SICI) and bathing suit ichthyosis (BSI) can be seen as well [6]. We discuss a case of ARCI with a pathogenic CYP4F22 mutation that presented at birth with a collodion membrane.

## Case presentation

**Figure 1:**
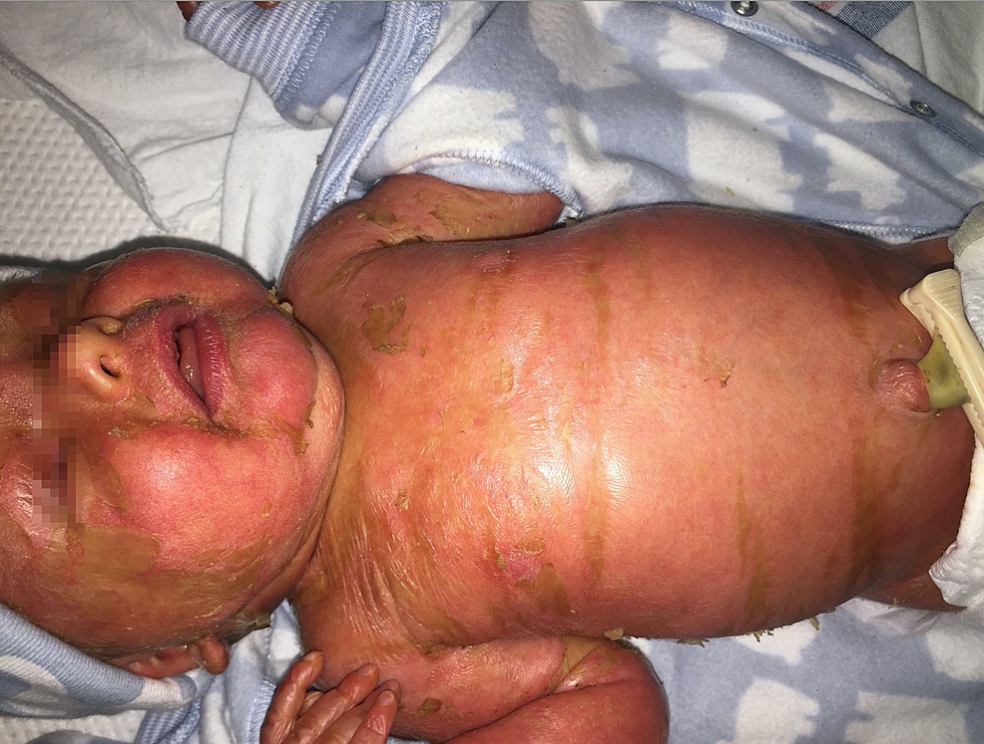
Newborn with collodion membrane at birth Involving the face, trunk, and extremities, there is an adherent, thick, yellow-brown, shiny membrane that has cracked open to reveal underlying erythroderma

A two-day-old male presented to the dermatology consult service after diffuse, thick scaling was noted at birth. On presentation, he was noted to have a focally adherent, thick, yellow-brown, shiny membrane that had cracked open revealing the underlying erythema (Figure 1). Nails and hair were noted to be normal and he had no ectropion or bullae. He initially presented with hypernatremia that resolved over several days. Clinically, the patient was diagnosed with congenital non-syndromic ichthyosis and was started on Aquaphor twice a day and placed in a humidified incubator set to 60% RH. Genetics was consulted and a Congenital Ichthyosis XomeDxSlice was ordered. Ophthalmology was also consulted, and they noted bilateral superficial punctate keratopathy for which Soothe ophthalmic ointment was started.

**Figure 2:**
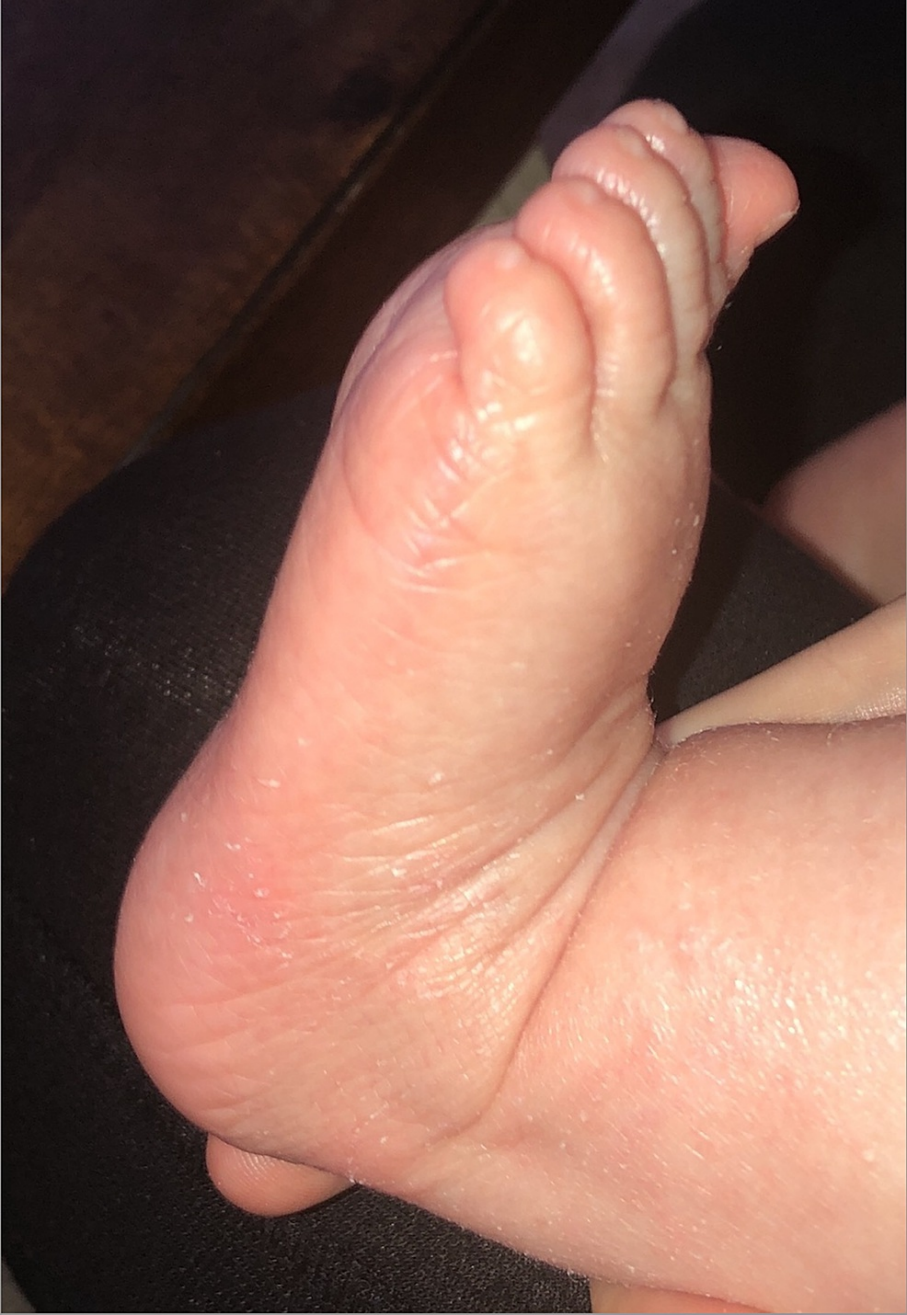
Patient's left foot There is a slight scale and hyperlinearity involving the left foot

Genetic testing revealed autosomal recessive, heterozygous CYP4F22-related congenital ichthyosis, and the patient was continued on a gentle skin-care regimen including Aquaphor twice a day and a daily bath with mild cleansers. The patient followed up at three months of age and showed overall improvement in the amount of erythema and scaling. A repeat exam revealed focal mild xerosis along with slight scale and hyperlinearity of the soles of the feet (Figure 2).

The patient followed up with ophthalmology for an episode of bilateral chalazion that resolved following the application of Tobradex ophthalmic ointment and oral antibiotic therapy. He continues to receive prophylactic warm compresses twice a week on his eyelids.

## Discussion

ARCI often presents at birth with a collodion membrane; it is caused by several different genetic mutations and can evolve into several different phenotypes. Only 8% of reported ARCI cases are due to a mutation in CYP4F22 [1]. It is critical to recognize congenital ichthyosis early as loss of the cutaneous barrier can lead to dehydration, electrolyte imbalances, infection, and penetration of external soluble materials [7]. During the neonatal period, supportive care is important and consists of emollients, a humidified incubator, and monitoring for hypernatremia.

The CYP4F22 gene is located on chromosome 19p13.12 and encodes a P450 cytochrome homolog of leukotriene B4 ω-hydroxylase [1]. CYP4F22 is the fatty acid ω-hydroxylase gene required for the production of acylceramide, which is an important lipid for the skin permeability barrier integrity [7]. This gene is also involved in the 12(R) lipoxygenase pathway, which is used for arachidonic acid metabolism and eicosanoid synthesis [8].

CYP4F22-related congenital ichthyosis can present with or without a collodion membrane at birth, depending on the type of gene mutation. Patients with a homozygous CYP4F22 missense mutation are not born with a collodion membrane, while patients with one or two mutations at the substrate-binding region may be born with a collodion membrane [6,8]. Most patients with a CYP4F22 mutation are born with marked erythroderma without a collodion membrane, hyperlinearity of palms and soles, and desquamation on the scalp, and develop a milder LI or CIE phenotype later in life [4]. This is consistent with our patient's case as he developed very mild focal scaling and hyperlinearity of the soles.

There have not been any reports of this mutation leading to ocular abnormalities such as bilateral chalazion and punctate keratopathy, which were noted in our patient. It is unclear if these are related to his underlying ichthyosis. Patients born with a collodion membrane who develop ocular abnormalities are four times more likely to have TGM1 mutations, but this was not the case with our patient [1].

Although we know that nonsense mutations in the ABCA12 gene causing LI or CIE and TGM1 mutations are responsible for a majority of LI cases, there is insufficient data on genotype/phenotype correlations for ARCI with CYP4F22 mutations in the literature [1]. Because CYP4F22-related congenital ichthyosis does not typically present with a collodion membrane and represents a small percentage of ARCI cases, this clinical presentation is extremely rare.

## Conclusions

We discussed a rare case of ARCI caused by a mutation in CYP4F22 that presented with a collodion membrane at birth. Through this case vignette, we would like to bring the clinicians' attention to the importance of recognizing congenital ichthyosis, since the loss of the cutaneous barrier can be life-threatening in a newborn.
